# Compensatory transporter upregulation facilitates retinal ganglion cell survival in glaucoma after MCT2 elimination

**DOI:** 10.3389/fcell.2026.1805959

**Published:** 2026-04-29

**Authors:** Kudakwashe P. Murinda, Autumn B. Morgan, Denise M. Inman

**Affiliations:** 1 Department of Pharmaceutical Sciences, UNT Health at Fort Worth, Fort Worth, TX, United States; 2 North Texas Eye Research Institute, UNT Health at Fort Worth, Fort Worth, TX, United States

**Keywords:** conditional knockout, GLUT3, MCT2, nicotinamide, SLC16A7, glaucoma

## Abstract

Metabolic dysfunction contributes to glaucoma progression, including through substrate availability and the presence and concentration of substrate transporters. Observations of metabolic substrate transporter loss in glaucoma, including loss of monocarboxylate transporter-2 (MCT2), have suggested there are serious implications for metabolic dysfunction on the health and survival of retinal ganglion cells (RGCs). In this study, we investigate whether MCT2 is necessary and sufficient for RGC survival *in vivo* and after ocular hypertension (OHT). We used an inducible conditional knockout (KO) mouse to remove MCT2 in Thy1-positive RGCs, then assessed RGC survival and function after OHT. MCT2 KO alone did not affect RGC density but did significantly reduce pattern electroretinogram amplitude. Upregulation of MCT1, MCT4, and GLUT3 transporters occurred as a result of MCT2 KO, suggesting that RGCs employ compensatory measures to meet their metabolic needs. Introducing oral nicotinamide (500 mg/kg/day) to test its ability to offset potential energy substrate insufficiency from MCT2 KO showed that nicotinamide was protective of RGC density for the MCT KO group but did not preserve RGC density or function for MCT2 KO + OHT. These data indicate RGCs are able to undergo compensatory adaptation to MCT2 KO with substrate transporter upregulation, which preserves their density but is not sufficient to fully preserve their function. Intervention that supplies metabolic intermediates can mitigate the loss of MCT2.

## 1 Introduction

Glaucoma, the leading cause of irreversible blindness worldwide (Williams and Casson, 2024), is characterized by the degeneration and ultimate death of retinal ganglion cells (RGCs). The clinical manifestation of glaucoma progression is initial loss of peripheral vision, followed eventually by total blindness. A major risk factor for the most common form of glaucoma is increased intraocular pressure (IOP), (Mao et al., 1991), a result of reduced aqueous humor outflow from the eye. Current glaucoma therapies decrease intraocular pressure but do not address the causes of the disease, nor other risk factors of glaucoma, such as metabolic deficiency, age, and genetics (Williams and Casson, 2024; Tielsch et al., 1991).

Cellular deprivation of energy substrates is implicated in most neurodegenerative diseases (Chen and Zhong, 2013; Vandoorne et al., 2018), including glaucoma (Williams and Casson, 2024; Harun-Or-Rashid et al., 2018; Harder et al., 2020). Consequences of this deprivation include compromised ATP levels, cellular dysfunction, and cell death. Diet or dietary supplement-based intervention has previously been shown to slow down or prevent cell death, even after pathology onset (Harun-Or-Rashid et al., 2018; Williams et al., 2017). The retina is especially vulnerable to metabolic dysfunction because it is a highly metabolic tissue (Harun-Or-Rashid et al., 2018) and is impacted by the bioavailability of essential energy metabolites such as glucose, pyruvate, and lactate, which implicates substrate transporters, including glucose transporters (GLUTs) (Khan et al., 2025) and monocarboxylate transporters (MCTs) (Harun-Or-Rashid et al., 2018), in glaucoma pathology. As a result, approaches towards glaucoma therapy that address metabolism in the RGCs may prove to be effective strategies for structural and functional survival.

RGCs are able to meet their metabolic needs by directly using glucose or lactate, an end product of glycolysis in astrocytes, which is taken up by the neurons via the MCTs (Brooks, 2018; Magistretti and Allaman, 2018). MCTs are widespread in the CNS, though MCT2 is concentrated on neurons, while MCT1 and MCT4 are found primarily on endothelial and glial cells, respectively (Simpson et al., 2007). Plasma membrane transporters such as the MCTs are translated in the cytoplasm and transported to the plasma membrane by chaperone proteins (Nakai et al., 2006; Wilson et al., 2005). MCT2 is associated with the chaperone embigin (Wilson et al., 2005; Ovens et al., 2010), without which the transporter would not be efficiently trafficked to the plasma membrane (Ovens et al., 2010). Each MCT has kinetics that determine the flow of substrates through it. MCT2 has a high affinity for lactate, so it often draws it in from the extracellular space, down its concentration gradient, while low-affinity MCT1 or 4 allow substrates to move down their concentration gradients and out of the cell (as in glia, where intracellular lactate concentrations are higher) (Bergersen, 2015). Utilizing lactate is purportedly more efficient than direct glucose use, as lactate only requires one enzymatic step to produce pyruvate, compared to glucose, which requires ten steps (Mason, 2017). In the RGCs, lactate is taken up via MCT2 (Murai et al., 2023). Previous studies have shown that MCT2 levels decline in the retina prior to glaucoma onset, and MCT2 overexpression is neuroprotective (Harun-Or-Rashid et al., 2020). Hence, cellular deprivation of energy substrates advances neuron death and disease progression and easing substrate delivery can mitigate metabolic stress.

In addition to rectifying transporter concentration decline in the retina, the observed inability to maintain nicotinamide adenine dinucleotide (NAD^+^), an important cofactor for redox reactions that is critical in energy metabolism, in glaucoma and other neurodegenerative diseases (Johnson and Imai, 2018), led to nicotinamide supplementation that proved to be an effective means by which to suspend neurodegeneration (Williams et al., 2017). NAD+ is needed to regulate the activity of dehydrogenases in multiple catabolic pathways, including glycolysis and fatty acid oxidation (Covarrubias et al., 2021). Previous studies have shown that observed pathophysiological conditions resulting from NAD + decline can be reversed by boosting NAD + levels through introducing its precursors (Vinten et al., 2025; Beltran et al., 2025). The salvage pathway is most utilized to regenerate NAD+ in the retina (Revollo et al., 2004). It uses nicotinamide phosphoribosyl transferase (NAMPT) to convert nicotinamide (NAM) into nicotinamide mononucleotide (NMN), which is then converted into NAD + by the enzymes NMNAT1-3 (Su et al., 2024). A phase 2 randomized clinical trial has shown that an oral combination of nicotinamide and pyruvate resulted in significant short-term improvement in patient visual function (De Moraes et al., 2022).

Our study was motivated to determine if MCT2 is necessary and sufficient for RGC survival in the retina and after ocular hypertension. Additionally, we examined whether oral nicotinamide intervention could mitigate any potential negative effect of eliminating MCT2, the key means by which RGCs obtain energetic substrates.

## 2 Materials and methods

### 2.1 Animals

MCT2^fl/fl^ mice were a generous gift from Dr. David Gozal at Marshall University. All mice were bred and housed at the University of North Texas Health Science Center, Fort Worth. Both sexes of mice were used. We crossed Tg (Thy1-cre/ERT2, -EYFP) HGfng/PyngJ (Jackson Laboratories Stock 12,708) and MCT2^fl/fl^ mice to generate Thy1-cre/ERT2:MCT2^fl/fl^ mice that were injected IP (75 mg/kg) with Tamoxifen (Sigma-Aldrich CAS # 10540–29–1, 10 mg/mL in sunflower seed oil) to express cre recombinase in Thy1-positive neurons and eliminate MCT2. Tamoxifen-injected mice were held for 30 days to allow for transporter turnover before any analysis was undertaken. Mice were maintained on a 12 h light cycle and had *ad libitum* access to standard rodent lab chow (Diet 5,058, PicoLab Mouse Diet 20 from Lab Diet). All animal procedures were reviewed and approved by the Institutional Animal Care and Use Committee (IACUC) at UNT Health under protocol number 2025–0020. The research was conducted in accordance with the guidelines set forth by the Association for Research in Vision and Ophthalmology, ensuring humane care and ethical treatment of all animals used in this study.

### 2.2 Intraocular pressure

Mice were anesthetized through inhalation of 2.5% isoflurane delivered through a vaporizer with oxygen. A Tono-Lab rebound tonometer (calibrated for mice; iCare) was used to take 10–20 measurements to calculate an average IOP; instrument-provided averages were not recorded. All IOP measurements were recorded within 3 min of anesthesia to avoid anesthesia-associated IOP decline (Harun-Or-Rashid et al., 2018; Chintalapudi et al., 2016). Baseline IOP was taken before ocular hypertension, then every 7 days after the procedure (for the relevant treatment groups) for 4 weeks.

### 2.3 Nicotinamide intervention

Nicotinamide (PanReac AppliChem A0959) was dissolved in water at a dose of 4 mg/mL, which translated to ∼500 mg/kg/day for each mouse based on an average water intake of 4 mL per day. Nicotinamide-supplemented water was provided in bottles protected from light that were changed every 3–5 days. Nicotinamide dosing commenced 7d prior to ocular hypertension and was maintained through 5 weeks total.

### 2.4 Pattern electroretinogram

RGC function was analyzed using the binocular snout pattern ERG (PERG animal research system, Jörvec Corp., Miami, FL, United States). Ketamine (100 mg/kg)-Xylazine (10 mg/kg) was administered IP to anesthetize the mice, which were kept on a heated stage during the procedure. Subcutaneous electrodes were placed on the nose (active), back of the head (reference), and tail (ground) of the mice. PERG was concurrently obtained from each eye in response to the contrast reversal of gratings produced by two LED screens run at different frequencies. The settings were as follows: Pattern mode: white-black; Pattern: 4 (4 white elements/4 black elements); Line filter: OFF; Gain: 10k; Low pass filter: 100 Hz; High pass filter: 1 Hz; Sweeps: 372 (block size of 31); Sampling time: 1 millisecond; Electrode montage: snout paradigm. The Jörvec software automatically identifies the P1 as the first reproducible positive peak after stimulus onset, and N2 for the main negative trough following P1. P1 is primarily associated with summed ON/OFF RGC pathway and somal activity, while N2 is dependent on RGC spiking and axonal function (Luo and Frishman, 2011). Latencies assist in peak and trough identification; P1 is generally ∼50 m while N2 is ∼95 m (Porciatti, 2007). Latency is measured from stimulus onset to the peak or trough of the deflection in question (P1 or N2). Amplitudes and latencies can vary, with glaucoma often showing a reduction in the P1 to N2 amplitude, and a slowing down of the emergence of the P1 and N2 deflections (Porciatti et al., 2007). The PERG amplitude is the P50 to N95 peak to trough amplitude (P1 to N2). PERG data were evaluated by collecting instrument measured amplitudes (P50-N95) and corresponding implicit times (latencies). Two successive runs per animal were used to plot the amplitudes and latencies.

### 2.5 Visual evoked potential

Visual pathway integrity from the retina to the visual cortex was assessed by measuring visual evoked potential (VEP). The Jörvec system used for PERG, including electrode placement as described above, was also used to obtain the VEP. P1-N1 waveforms were analyzed to get the amplitude (peak-to-peak mV) and latencies (Porciatti et al., 2002). P1 reflects activation in visual cortex, a basic index of sensory encoding visual input, while the N1 is later stage early visual processing (You et al., 2011).

### 2.6 Ocular hypertension

Mice were anesthetized using 2.5% isoflurane as previously described. Tropicamide (0.05%) was topically applied to the eye to dilate the pupil, followed by Proparacaine (0.05%) to locally anesthetize the cornea. Ocular hypertension was induced through an intracameral injection (2.0 μL) of magnetic microbeads (a 1:2 mixture of 4 and 8 μm-diameter beads; Bangs Laboratories) into the anterior chamber using a glass-pulled micropipette. Magnetic microbeads were distributed along the iridocorneal angle using a neodymium magnet to draw them into the trabecular meshwork (Jassim and Inman, 2019). Both eyes of each mouse in the OHT groups were injected with microbeads. Mice whose eyes reached an IOP integral of >150mmHg-days were used. IOP integral was calculated by summing the mmHg above baseline experienced by the eye over the 4 weeks of the post-OHT period.

### 2.7 Immunofluorescence and microscopy

Mice were euthanized through an overdose of sodium pentobarbital and sodium phenytoin (300 mg/kg, 50 mg/kg; Euthasol) by IP injection. Eyes were freshly dissected after transcardial perfusion with 0.1 M PBS, then 4% paraformaldehyde (PFA) for tissue fixation. Eyes were placed in 4% PFA for 30 min and then moved to 30% sucrose with 0.02% sodium azide at least overnight before further processing.

For eyes intended for sectioning, the cornea and lenses were removed from the globes, which were then embedded in OCT and frozen in liquid-nitrogen-cooled isopentane before sectioning (10 μm) using a cryostat (Leica). Retina sections were subsequently used for immunohistochemistry using the antibodies listed in Table 1.

**TABLE 1:** Primary Antibodies used for Immunohistochemistry.

Antibody	Dilution	Company	Host	Catalog number
RBPMS	1:250	GeneTex	Rabbit	GTX118619
RBPMS	1:250	Phosphosolutions	Guinea pig	1832-RBPMS
MCT2	1:100	Santa cruz biotechnology	Mouse	sc-271093
MCT1	1:100	Santa cruz biotechnology	Mouse	sc-365501
MCT4	1:100	Santa cruz biotechnology	Mouse	sc-376465
Embigin	1:100	Protein tech	Rabbit	13946-1-AP
GLUT3	1:100	Santa cruz biotechnology	Mouse	sc-74497
P2X7R	1:250	Novus biologicals	Goat	NBP1-37775
TNFα	1:50	Abcam	Mouse	ab1793

For eyes intended for whole mount retina, after enucleation, the cornea and lens were carefully removed. A slit was cut into the sclera to ease the removal of the retina from the globe, after which four relaxation cuts were placed in the retina. Vitreous humor was removed, followed by cryoprotection in 30% sucrose in 0.1M PBS, then a sequence of three freeze-thaws to promote antibody penetration. Whole-mounted retinae were immunolabeled with an antibody against RNA-binding protein with multiple splicing (RBPMS) to label RGCs.

Whole mounts and sections were rinsed in PBS, blocked with 5% donkey serum and 0.4% Triton X-100 in 1X PBS, then incubated with primary antibodies prepared in the blocking buffer for 2 days (whole mounts) or overnight (sections) at 4 °C. Tissue and sections were rinsed in PBS, then blocked briefly in 5% DKS +0.4% Triton in PBS. Secondary antibodies prepared in the same block were added and incubated overnight at 4 °C (whole mount) or for 2 h (sections). Whole retinae were mounted on glass slides, RGC-side up, then, like sections, cover slipped in DAPI-FluoromountG (Southern Biotech) and imaged using a Zeiss LSM-880 confocal microscope. See Table 1 for the list of antibodies used for Immunohistochemistry.

### 2.8 IHC quantification

Immunolabeling was quantified in the ganglion cell layer (GCL) using ImageJ. Raw, uncompressed TIFF images were used. The image channels were split, and the RGC marker channel (RBPMS) was analyzed first. The RBPMS channel was converted to 8-bit (image > type). The color threshold was adjusted (adjust > threshold) until the GCL was highlighted. A selection of the desired layer was created, and the unwanted inside of the selection was cleared (edit > selection > create selection > clear). The selection was saved (file > save as > selection). After this, the desired channel to be measured was selected, and the saved GCL selection was opened and superimposed. The outside of the wanted selection was cleared (edit > clear outside) and the selected area measured (analyze > measure). The mean fluorescence intensity was used for our statistical analysis. At least 3 retinae were analyzed per group, with five sections per retina, for the different proteins.

### 2.9 RGC quantification

Unbiased stereological quantification of RBPMS-positive RGCs in whole-mounted retina using a ×20 objective was performed using the optical fractionator module within StereoInvestigator (MicroBrightfield Bioscience, Williston, VT, United States). A 50 × 50 μm counting frame was used across approximately 50–60 sites (10%), with RBPMS-positive RGCs quantified at each sampling site. The coefficient of error (Schmitz-Hof) was maintained at 0.05 or below, ensuring a sufficient sampling rate (Harun-Or-Rashid et al., 2018).

### 2.10 Quantitative real-time PCR

mRNA was extracted from flash-frozen retinas using a Qiagen RNeasy kit (Cat# 74134) according to the manufacturer’s instructions. In brief, homogenization of retinas in RLT buffer followed by binding of mRNA to a capture column with subsequent washes allowed for cleaning then elution of mRNA. The isolated mRNA was converted to cDNA using the Verso cDNA Synthesis Kit (Thermo Fisher Scientific, AB-1453/B) for stable storage and analysis using real-time quantitative qPCR using TaqMan assay probes (Table 2) and a Quant Studio 5 PCR System instrument (Applied Biosystems). β-actin was the housekeeping gene, and a control (naive) sample was the endogenous control. Four biological replicates were used for each transcript, and each run in triplicate. See Table 2 for list of TaqMan Assays used for qPCR and their catalogue numbers.

**TABLE 2:** TaqMan Assays used for qPCR.

mRNA	TaqMan assay
*Slc16a7 (MCT2)*	Mm004411442_m1
*MPC1*	Mm00834592_g1
*GLUD1*	Mm00492353_m1
*LDH-a*	Mm01612132_g1
*LDH-b*	Mm05874166_g1
*PFKP*	Mm00444792_m1
*6-phosphofructo-2-kinase/fructose-2,6-biphosphatase 3*	Mm00504650_m1
*Slc2a3 (Glut3)*	Mm00441483_m1
*RBPMS*	Mm00803908_m1
*Slc16a1 (MCT1)*	Mm01306379_m1
*Slc16a3 (MCT4)*	Mm00446102_m1
*Actb (β-Actin)*	Mm02619580_g1

### 2.11 Protein analysis

Flash frozen retinas were sonicated for homogenization in T-PER (Tissue Protein Extraction Reagent, Thermo Fisher Scientific Product # 78510) with 1 μL of 100× Halt Protease and Phosphatase Inhibitor (Thermo Fisher Scientific, #1861280), then centrifuged for 15 min at 10,000g at 4 °C. The supernatant was collected into a new tube and used for protein analysis. Total retinal protein concentration was determined using Pierce™ BCA Protein Assay Kit (Thermo Fisher Scientific # 23225) with absorbance read at 562 nm using a Cytation 5 (Biotek) plate reader. Protein analysis was performed via capillary-based electrophoresis immunoassay using the Protein Simple Jess instrument (Biotechne); see Table 3 for the list of proteins analyzed.

**TABLE 3:** Antibodies used for Capillary Electrophoresis.

Antibody	Dilution	Company	Host	Catalog number
LDH-a	1:50	Novus biologicals	Rabbit	NBP1-48336
MCT2	1:25	Santa cruz	Rabbit	Sc-50323
Tom20	1:25	Cell signaling	Rabbit	42406-S
pAMPK (pThr172)	1:25	Novus biologicals	Rabbit	NBP1-74502
AMPK	1:100	Novus biologicals	Mouse	NBP2-22127

### 2.12 ATP quantification

ATP levels in the whole retina were measured using an ATP measurement kit (Thermo Fisher Cat # A22066), which uses luciferase bioluminescence to indirectly quantify ATP. Supernatant from flash frozen retinae that were homogenized by sonication in T-PER (tissue protein extraction reagent; ThermoFisher) with 100X Halt Protease (Pierce) as described above (in protein analysis) was used. The ATP values were normalized using the protein sample concentrations as determined by a BCA (Bicinchoninic Acid) protein assay (Pierce) read on a Cytation5 (BioTek) at 562 nm.

### 2.13 Fluorescent activated cell sorting

Mouse eyes were enucleated after CO_2_ euthanasia and cervical dislocation. Retinae were removed and dissociated by incubation in papain (9 U) in 0.1 M PBS for 30 min at 37 °C, followed by trituration. The materials were prepared according to the protocol described in (Chintalapudi et al., 2016). Flow cytometry was performed using a Sony SH800 on retinal cell suspensions to isolate RGCs, which were selected for using conjugated antibodies for the Thy1.2 antigen (anti-CD90.2 Alexa Fluor-700) and against CD48 (anti-CD48 PE-Cy7), CD15 (anti-CD15 PE), and CD57 (anti-CD57, which was conjugated with a Brilliant violet 421-tagged secondary antibody) (Chintalapudi et al., 2016). After retinal cell dissociation, the cells were treated with 1.0 μg of anti-CD16/CD32 per 1.0 × 10^6^ cells in 100 μL to minimize nonspecific binding and inhibit endocytosis, phagocytosis, and antigen presentation. The following diode lasers were used: 488 nm blue, 630 nm red and 405 nm violet. Dissociated cells were maintained at 4 °C, and unlabeled murine retinal cells were used as controls. Individual samples were labeled with antibodies specific for each of the individual cell surface markers and sorted.

### 2.14 *In situ* hybridization

Retina tissue was fixed, embedded, and sectioned as described above. Tissue was processed following the protocol from the fixed-frozen tissue sample preparation and pretreatment section in the ACD RNAscope Multiplex Fluorescent Reagent Kit v2 User Manual. The slides were baked in the RNAscope oven at 40 °C. Slides were dehydrated in 50%, 70% and 100% ethanol in sequence. Slide sections were covered with RNAscope H_2_O_2_ for 5–10 min at room temperature and target retrieval was completed by submerging slides in target retrieval reagent heated to ∼100 °C for 5 min then washing for 3–5 times in distilled water. Protease digestion was done by applying RNAscope Protease III on the sections, incubating for 30 min at 40 °C then washing twice with distilled water. Probes were hybridized by adding probe mixtures to the slides at the appropriate dilution and incubating at 40 °C for 2 h. The appropriate fluorophores for the relevant channels were added, and the slides were counterstained and mounted with anti-fade mounting media. Our target probes were RBPMS (Mm-Rbpms REF 527231) and MCT2 (Mm-Slc16a7-C2 REF 509921-C2). The slides were imaged using a Zeiss LSM 880 confocal microscope.

### 2.15 Experimental design and statistical analysis

Data were analyzed using GraphPad Prism v.9 (La Jolla, CA, United States). Statistical analyses were performed after evaluating normality using the Shapiro–Wilk test and then choosing the appropriate parametric or non-parametric tests. Repeated measures ANOVA was used to analyze IOP data. Two-way ANOVA was used to analyze the datasets that included the nicotinamide data. Unpaired, two-tailed *t*-tests were used to compare differences across groups within an outcome measure; *p* < 0.05 was considered statistically significant. A two-way ANOVA (mixed effects analysis) was used to analyze the PERG and VEP data in order to conform with best practices regarding use of techical replicates because two rounds of PERG and VEP were included per mouse for Initial and Final values, and both eyes were included. Individual eyes were not considered independent values for the PERG and VEP data. We tested for outliers using the ROUT test on Graphpad Prism with a Q value of 1%, with any datasets excluding outliers indicated in figure legends.

## 3 Results

### 3.1 MCT2 knockout

**FIGURE 1:**
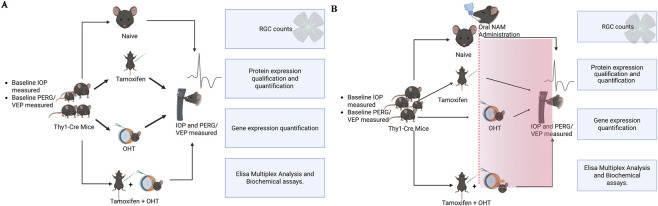
Schematic of the experimental design. **(A)** Thy1-ERT2-cre: MCT2^fl/fl^ mice were used to conditionally knock out (KO) MCT2 in Thy1-positive retinal ganglion cells (RGCs). Tamoxifen-induced cre recombination and elimination of MCT2 commenced 1 month prior to ocular hypertension (OHT) to ensure sufficient time for endogenous MCT2 turnover and complete elimination of the transporter in Thy1-positive RGCs. In part one of the study **(A)**, after baseline IOP and PERG/VEP were obtained, mice were divided into four treatment groups (Naïve, KO, OHT, and KO + OHT). Bilateral OHT was performed (see Methods) in the OHT and KO + OHT groups and maintained for 4 weeks. IOP was measured weekly. Prior to sacrifice, PERG/VEP was measured again. Tissue samples were collected for data collection and analysis. **(B)** In part two of the study **(B)**, a nicotinamide (NAM) intervention using the same groups and experimental design as in **(A)** transpired. Nicotinamide dosing commenced 7d prior to bilateral OHT induction. Analysis included quantification of RGC density, protein, gene expression, and cytokine levels across the different treatment groups. Schematic was created in BioRender. Murinda, **(K)** (2026) https://BioRender.com/4h08gzj.

This study comprised two parts, with an examination of the impact of knocking out MCT2 in the Thy1-positive RGCs with or without ocular hypertension (OHT) (Figure 1A, Experimental Design). Mice were injected with tamoxifen (see Methods) 4 weeks prior to commencement of experiments in order to allow for complete turnover and elimination of MCT2 in Thy1-positive RGCs via cre-recombinase mediated knockout (KO). Part two of the study included treatment of MCT2 knockout (KO) mice for 5 weeks with nicotinamide, with or without OHT (Figure 1B). Longitudinal measures collected included intraocular pressure, pattern electroretinogram (PERG), and visual evoked potential (VEP). Outcomes included RGC quantification and protein and mRNA analysis (Figures 1A,B).

**FIGURE 2:**
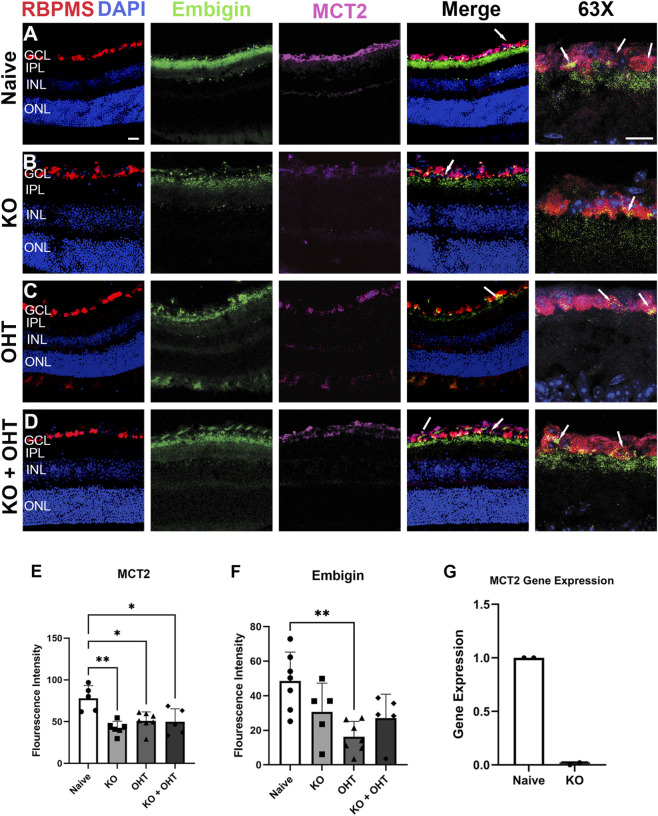
MCT2 and embigin distribution in the retina. **(A-D)** Immunofluorescence of Naïve **(A)**, KO **(B)**, OHT **(C)**, and KO + OHT **(D)** showing localization of MCT2 and embigin in the retina. Panels second from the right are merged images of DAPI (blue), RBPMS (red, RGCs), embigin (green), and MCT2 (magenta). The rightmost panels are 63X + 2X optical zoom images of the inner retina. Arrows point to RGCs where MCT2 and embigin are colocalized. The Naïve group **(A)** shows more prominent expression of MCT2 compared to the KO **(B)** and KO + OHT **(D)** groups, confirming that MCT2 was conditionally knocked out in the GCL of the knockout groups. Decreased MCT2 immunolabel in the GCL in the OHT group **(C)** corroborates previous data of a decline in MCT2 with OHT (Harun-Or-Rashid et al., 2020). Scale bar in **(A)** on left = 50 µm and on right = 25 µm for the 63X + 2X optical zoom. Embigin declines can be observed in the KO **(B)**, OHT **(C)**, and KO + OHT **(D)** groups compared to the Naïve **(A)** group. **(E)** and **(F)** show immunolabeling quantification of MCT2 in the GCL and embigin expression in the GCL + IPL, respectively. **(E)** The Naïve (n = 5) group had significantly more MCT2 immunolabel fluorescence intensity than the KO (n = 6, **p = 0.0032), OHT (n = 7, *p = 0.0343), and KO + OHT (n = 5, *p = 0.0329) groups. **(F)** The Naive group (n = 7) had significantly more embigin immunolabel (fluorescence intensity) than the OHT group (n = 7, **p = 0.002) but no statistical difference with the KO (n = 5) and KO + OHT (n = 5). **(G)** RGCs isolated from Naïve and KO mice using fluorescent-activated cell sorting (FACS) had mRNA isolated and analyzed using qPCR. MCT2 was not detected by real-time qPCR from isolated KO RGCs, confirming cre-recombinase-mediated KO.

To demonstrate the success of MCT2 KO in the Thy1-positive RGCs, we undertook immunolabeling for MCT2 (Figures 2A–D), its quantification by fluorescence intensity, and that of embigin in Figures 2E,F, and fluorescent activated cell sorting (FACS) of RGCs followed by qPCR for MCT2 (Figure 2G). Figures 2B,E documents a significant decline in MCT2 in the ganglion cell layer (GCL) of the KO, OHT, and KO + OHT groups (p = 0.0032 for KO and p = 0.0343 for OHT and p = 0.0329 for KO + OHT) compared to Naive. The significant decline in MCT2 in the OHT group corroborates previous data that documented glaucoma-associated MCT2 loss (Harun-Or-Rashid et al., 2020). The MCT2 chaperone protein embigin was also analyzed. MCT2 forms a complex with embigin and will not be efficiently trafficked to the plasma membrane without it (Ovens et al., 2010). Additionally, disruption of embigin reduces MCT2 expression (Ovens et al., 2010). Embigin shows a complementary decline along with MCT2, but this decline is only significant in the OHT group (p = 0.0020; Figure 2F). RNA from FACS-isolated RGCs was analyzed using qRT-PCR for MCT2 transcript; no MCT2 was detected in KO RGCs (Figure 2G).

### 3.2 *In situ* hybridization for MCT2

**FIGURE 3:**
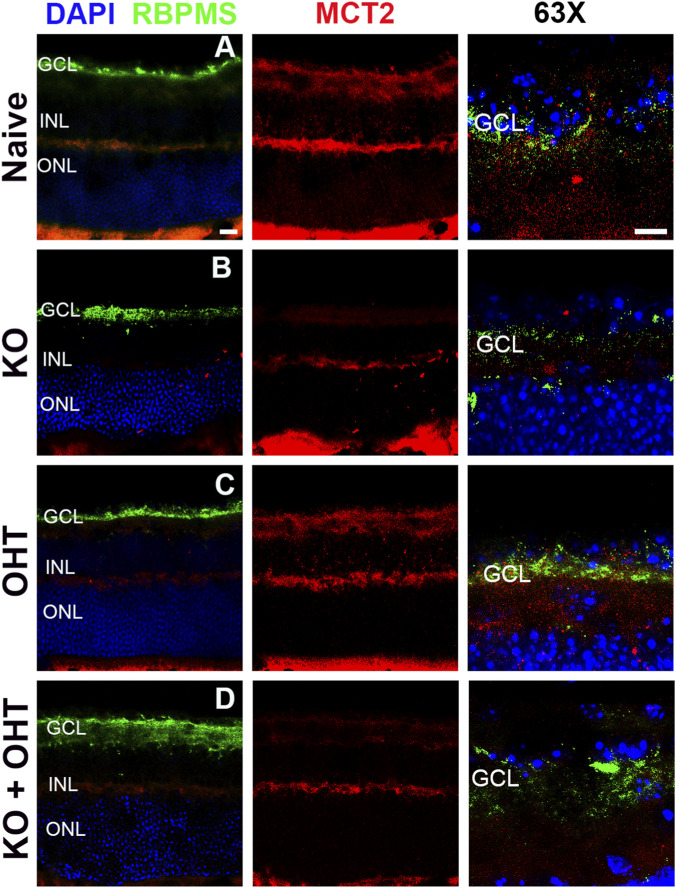
*In situ* hybridization using the RNAscope (see Methods). **(A)** Oligonucleotide probes for RBPMS (green) and MCT2 (red) showed greater MCT2 transcript in the Naive group in the GCL and upper IPL. **(B)** The KO group had no MCT2 transcript in the GCL, while maintaining MCT2 in the OPL. **(C)** The OHT group had MCT2 transcript reminiscent of Naive, with some dissipation in the OPL. **(D)** The KO + OHT group showed no MCT2 transcript in the GCL while slight dissipation of the transcript in the OPL as compared to Naive. DAPI (blue) labels nuclei. Scale bars = 50 µm.

MCT2 mRNA levels were measured by *in situ* hybridization (Figure 3) in order to confirm the elimination of MCT2 in the RGCs. MCT2 mRNA was prominent in the GCL, inner IPL, and photoreceptor outer segments (Figure 3A) in Naive retina (red fluorescence). The innermost portion of the IPL is the location of ON RGC dendritic arbors, suggesting a high concentration of MCT2 in ON RGC dendrites. MCT2 transcript was undetectable in the GCL of KO retina (red; Figure 3B) but was evident in the OPL. The OHT group showed MCT2 transcript not unlike Naive, though the expression in the OPL was not as concentrated as Naive (compare Figures 3A–C). KO + OHT led to notable decreases in MCT2 transcript in GCL (Figure 3D).

### 3.3 RGC density and function

**FIGURE 4:**
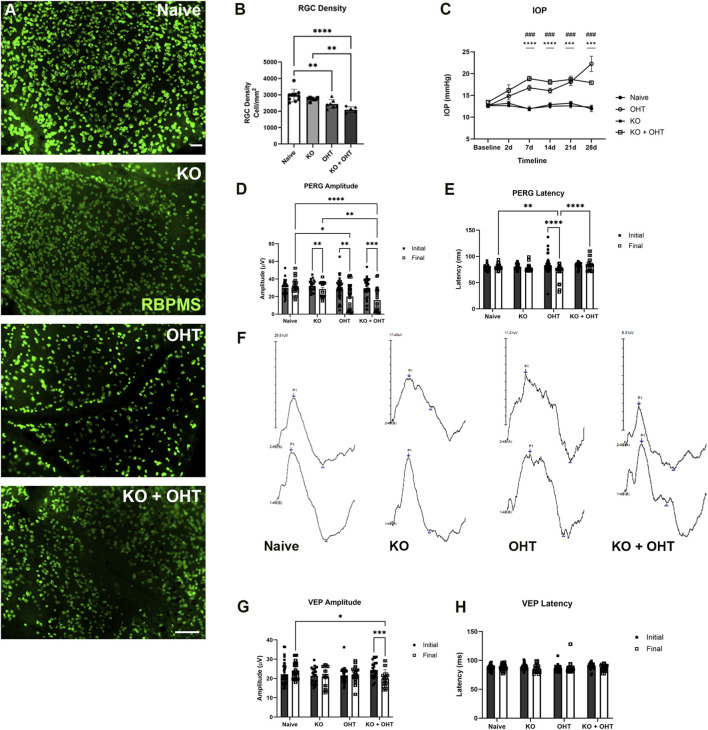
Retinal Ganglion cell survival and function. **(A)** Whole mount retinae immunolabeled with RBPMS (green). Images show retina roughly 700–1000 microns from the optic nerve head. Scale bar = 50 μm. **(B)** RGC density quantified using unbiased stereology. MCT2 KO (n = 7) alone did not significantly decrease RGC density. A significant decline in RGC density was observed in the OHT group (n = 7, p = 0.0046) and KO + OHT (n = 5, p < 0.0001) compared to Naive. KO + OHT RGC density was significantly lower than KO alone (p = 0.0026). **(C)** IOP was significantly increased over baseline in the OHT and KO + OHT groups (***p < 0.0001 and ***p = 0.0002). **(D)** RGC function was measured using pattern electroretinogram (PERG) amplitude. The final PERG amplitude, measured 4 weeks after Initial for all groups, showed a significant amplitude decline in the KO (n = 12, **p = 0.0057), OHT (n = 16, **p = 0.0031), and KO + OHT (n = 12, ***p = 0.0003) groups. In addition, Final OHT PERG amplitude was significantly lower than Naive (n = 18, *p = 0.0101); KO + OHT was significantly lower than KO (**p = 0.0010); and KO + OHT was significantly lower than Naive (****p < 0.0001). **(E)** PERG latency in Naive for the Final measure was significantly different than OHT Final (**p = 0.0011); OHT Initial was significantly greater than Final (****p < 0.0001); and KO + OHT Final was significantly greater than OHT Final (****p < 0.0001). **(F)** Example PERG traces for each of the Naive, KO, OHT, and KO + OHT groups. Left (top) and right eyes (bottom) are shown. **(G)** Visual pathway function was measured using Visual Evoked Potential (VEP) amplitude **(G)** and latency **(H)**. Unlike PERG amplitude, VEP amplitude was not impacted by MCT2 KO alone. Naive Final VEP amplitude was significantly greater than Final KO + OHT VEP amplitude (*p = 0.0379). Initial and Final VEP amplitude for the KO + OHT group was significantly decreased (***p = 0003). **(H)** There were no statistical differences across groups or time point (Initial and Final) for VEP latency. VEP n were the same as listed for PERG.

A significant decline in RGC density was observed after the induction of OHT (p = 0.0049, one-way ANOVA), Figures 4A,B. MCT2 KO alone did not impact RGC density, as shown by no statistical difference in RGC density between Naive and MCT KO groups (Figure 4B). Both the OHT and KO + OHT groups experienced a significant loss of RGCs compared to Naive retina (p = 0.0046 and p < 0.0001, respectively; Figure 4B). The KO + OHT retinae also had significantly fewer RGCs than the KO group alone (p = 0.0026; Figure 4B). The magnetic microbead model induced a significant increase in IOP over 4 weeks for the OHT and KO + OHT groups, (Figure 4C; p = 0.0005 for OHT and p = 0.0003 KO + OHT groups, by repeated measures ANOVA). Pattern electroretinogram (PERG), which measures the magnitude of RGC electrical response to a patterned visual stimulus, and latency, which measures the timing response after stimulus onset, were obtained in all groups at baseline (Initial) and 4 weeks after OHT induction (Final) (Figures 4D,E). A significant decline in PERG amplitude (Figure 4D) was recorded between Initial and Final PERG in the KO (p = 0.0057), OHT (p = 0.0031), and KO + OHT (p = 0.0003) groups. For the KO group, the Initial PERG amplitude measures occurred prior to MCT2 KO, so Final PERG amplitude represents the inner retina electrical response as a result of loss of MCT2. In comparison to the Naive group, a decline in Final PERG amplitude was seen in the OHT (p = 0.0101) and KO + OHT (p < 0.0001) groups (Figure 4D). A decline in Final PERG latency (Figure 4E) was observed in the OHT group (p < 0.0001) compared to Initial latency. Example PERG traces are shown for each of the Naive, KO, OHT, and KO + OHT groups in Figure 4F. Final VEP amplitude (Figure 4G) was significantly decreased in the KO + OHT group (p = 0.0379) compared to Naive, and Initial VEP amplitude in KO + OHT was significantly higher compared to Final (p = 0.0003). There were no statistical differences across groups and time points for VEP latency (Figure 4H).

### 3.4 Transporter upregulation

**FIGURE 5:**
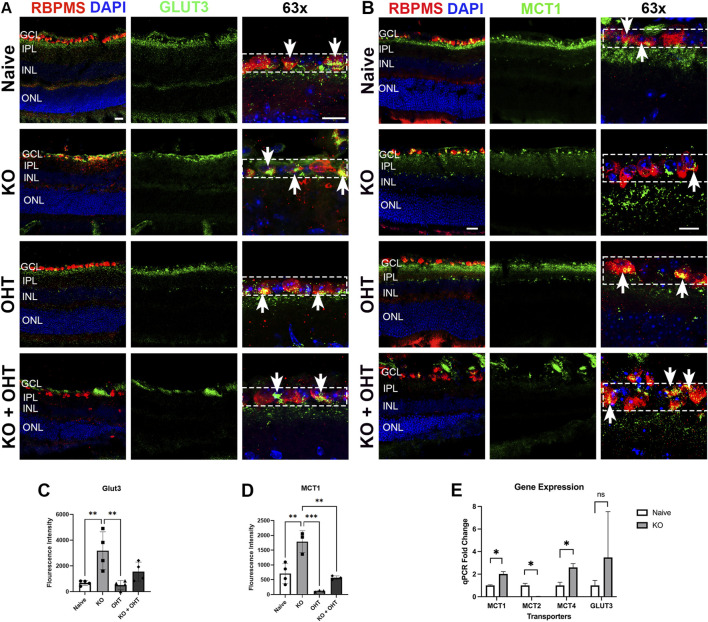
Compensatory upregulation of transporters observed after MCT2 KO in retina. **(A)** Immunolabeling for GLUT3 (green), an insulin-independent glucose transporter preferentially expressed by neurons, showed strong label across all groups. RBPMS (red) and nuclear stain DAPI (blue) enable colocalization of GLUT3 with RGCs in the GCL. Arrows show colocalization of GLUT3 and RBPMS in the GCL layer. **(B)** MCT1 immunolabeling indicated strong protein expression across groups in the GCL. RBPMS (red) and nuclear stain DAPI (blue) enable colocalization of MCT1 with RGCs. Arrows show colocalization of GLUT3 and RBPMS in the GCL layer **(C)** Quantification of fluorescence intensity of GLUT3 in the GCL (regions of interest denoted by dotted line boxes) indicates significantly higher GLUT3 in the KO (n = 4) mouse retina compared to Naive (n = 4, **p = 0.0059) and OHT (n = 4 **p = 0.0037) groups but not the KO + OHT (n = 4) group. **(D)** Quantification of MCT1 immunolabeling in the GCL layer (regions of interest denoted by dotted line boxes) showed significantly higher MCT1 in KO (n = 4) compared to Naive (n = 4, **p = 0.0022), OHT (n = 3 ***p = 0.0001), and KO + OHT groups (n = 3, **p = 0.0014). **(E)** Gene expression levels from a pure population of RGCs isolated using FACS showed that the MCT2 KO group had no detectable MCT2 transcripts compared to Naive (n = 3, *p = 0.0326) and higher expression of MCT1 (n = 3, *p = 0.0321) and MCT4 (n = 3, *p = 0.0233) compared to Naive. GLUT3 was highly variable, with no statistical difference (ns, p = 0.604).

Elimination of a major means by which RGCs take up pyruvate, lactate, and ketone bodies (MCT2) would be anticipated to impact other energy substrate transporters. To wit, we observed significant upregulation of both GLUT3, an insulin-independent glucose transporter expressed on neurons (Figures 5A,C), and MCT1, a monocarboxylate transporter with less affinity for pyruvate and lactate compared to MCT2 (Figures 5B,D) in the GCL. Quantification of immunolabel was for the ganglion cell layer only (GCL; dotted line boxes in left panels of Figures 5A,B). In some cases, immunolabeling was prominent in the inner IPL, the strata of arborization for ON-RGCs. These areas were excluded from the quantification in favor of capturing somal transporter expression since the labeling in the ON-RGC synaptic region could not be ascribed solely to RGCs. MCT2 KO retinae had significantly more GLUT3 immunolabeling (Figure 5A, with quantification in Figure 5C; p = 0.0059) and MCT1 immunolabeling (Figure 5B, with quantification in Figure 5D; p = 0.0022) in the GCL than Naïve retinae. The KO group also had statistically significantly more GLUT3 in the GCL than the OHT group (Figure 5C; p = 0.0037). MCT1 was significantly higher in the KO GCL than the Naive (p = 0.0022), OHT (p = 0.0001) and KO + OHT groups (p = 0.0014) as well (Figure 5D). To further characterize the changes in transporters after MCT KO, we isolated RGCs using FACS (see Methods) and then isolated mRNA for subsequent qRT-PCR. The KO group exhibited no detectable MCT2 gene expression in RGCs, which was significantly lower than Naive (p = 0.0326); significantly higher MCT1 (p = 0.0321) and MCT4 (p = 0.0233) gene expression was observed in the MCT2 KO compared to Naive (Figure 5E). GLUT3 expression in MCT2 KO compared to Naive was too variable for a statistical difference (p = 0.604).

By immunohistochemistry (Supplementary Figure S1A-D) and quantification of immunofluorescence intensity (Supplementary Figure S1E), MCT4 did not vary in a statistically significant way across groups. There was a noticeable decline in MCT4 with OHT (Supplementary Figure S1E); however, the immunolabeling was variable enough across groups that this was not statistically different.

Analysis of whole retina lysates showed a significant upregulation of MCT2 protein in the KO + OHT group (Supplementary Figure S2A). This MCT2 represents expression in cells other than Thy1-positive RGCs which we have otherwise shown (Figures 2A,E,G; Figure 3B). Interestingly, *in situ* hybridization for MCT2 showed very low transcript levels in KO + OHT, particularly in the GCL, despite the whole retina protein upregulation in this group (Figure 3C). We also evaluated LDH-a protein (Supplementary Figure S2B), the ratio of pAMPK to AMPK (Supplementary Figure S2C), and Tom20 protein (Supplementary Figure S2D) using whole retina lysates using capillary electrophoresis, finding no differences in these proteins across groups. LDH-a catalyzes the conversion of pyruvate to lactate, so might have been impacted by substrate availability, while pAMPK/AMPK ratio would be expected to increase in conditions of limited ATP supply. Tom20 is a proxy for mitochondria number. MCT2 KO mouse retina did show significantly higher Tom20 (p = 0.0441) compared to Naive (Supplementary Figure S2D).

### 3.5 Metabolic transcript changes

**FIGURE 6:**
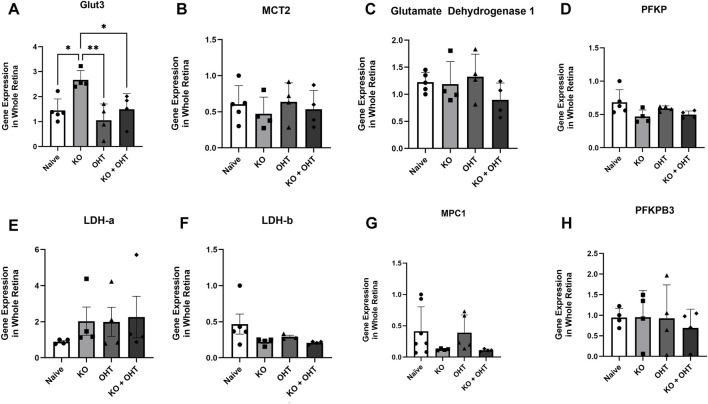
Quantitative RT-PCR for whole retina. **(A)** The KO (n = 4) group retina had statistically higher glucose transporter 3 (GLUT3) gene expression compared to the Naïve (n = 5, p = 0.0211), OHT (n = 4, p = 0.0044), and KO + OHT (n = 4) groups (p = 0.0358). No significant differences were observed in gene expression across whole retina for **(B)** monocarboxylate transporter 2 (MCT2), **(C)** Glutamate Dehydrogenase 1, **(D)** Phosphofructokinase (PFKP), **(E)** lactate dehydrogenase A, **(F)** lactate dehydrogenase B, **(G)** mitochondrial pyruvate carrier (MPC1) and **(H)** 6-phosphofructo-2-kinase/fructose-2,6-biphosphatase 3 (PFKFB3) among the groups. Subject numbers: For MCT2, Glutamate Dehydrogenase, PFKP, LDH-a and LDH-b; Naïve n = 5, KO n = 4, OHT n = 4 and KO + OHT n = 4. For MPC1: Naïve n = 7, KO n = 4, OHT n = 5 and KO + OHT n = 4. For PFKPB3: Naïve n = 4, KO n = 4, OHT n = 4 and KO + OHT n = 4.

Quantitative PCR of whole retina was undertaken to ascertain if there would be evidence of overall metabolic shifts through transcript changes because loss of MCT2 could result in decreased movement of carbon into the TCA cycle from decreased lactate, pyruvate, and ketone bodies. In the KO retina, there was significant upregulation of Slc2a3 (Glut3) compared to Naive (p = 0.0211), OHT (p = 0.0044), and KO + OHT (p = 0.0358) groups (Figure 6A), echoing the increased GLUT3 protein in the GCL. Glutamate dehydrogenase 1 (Glud-1) is a mitochondrial enzyme that facilitates the conversion of glutamate to α-ketoglutarate, which feeds into the TCA cycle. Its increase can represent a stress response to metabolic demands. Phosphofructokinase (PFKP), and 6-phosphofructo-2-kinase/fructose-2,6-biphosphatase 3 (PFKFB3), both important enzymes in glycolysis, were examined to gauge whether shifts in glycolysis would be evident (Chiba et al., 2025). LDH-a and LDH-b were examined to determine if conversion of pyruvate to lactate (LDH-a), or lactate to pyruvate (LDH-b) might have resulted from the potential changes in substrate availability (Lin et al., 2022). Mitochondrial Pyruvate Carrier 1 (MPC1) transports pyruvate into the mitochondria; its upregulation would be indicative of increased oxidative phosphorylation (Sun et al., 2025). There were no retinal changes across groups for these transcripts: MCT2, glutamate dehydrogenase, phosphofructokinase, lactate dehydrogenase-a, lactate dehydrogenase-b, mitochondrial pyruvate carrier, and 6-phosphofructo-2-kinase/fructose-2,6-biphosphatase 3 (PFKFB3); see Figures 6B–H.

As an overall assessment of retinal health, we measured ATP in whole retina across Naive, KO, OHT, and KO + OHT groups. We did not observe significant differences in retinal ATP across groups (Supplementary Figure S3A) which corroborates the absence of pAMPK/AMPK increases observed in Supplementary Figure S2C. While considering the possibility of ATP release if ATP levels were to vary across groups, we also immunolabeled retinal sections with P2X7 receptor and TNFα and quantified fluorescence intensity (Supplementary Figure S3B,C). P2X7 varied across groups, but not in a statistically significant way (Supplementary Figure S3B). TNFα was significantly increased in the OHT group compared to KO + OHT (p = 0.0247, Supplementary Figure S3C).

### 3.6 Nicotinamide improves RGC function after OHT for the MCT2 KO

**FIGURE 7:**
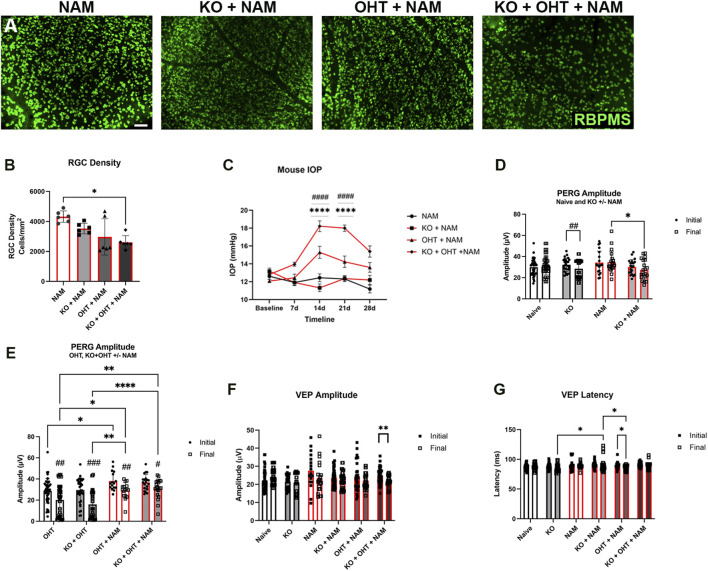
Nicotinamide was administered orally in part two of this study as a prophylactic measure against the impact of MCT2 KO. **(A)** Whole mount retinae immunolabeled with RBPMS (green), labeled by group. Images show retina roughly 700–1000 microns from the optic nerve head. PERG and VEP were measured for the NAM (n = 10), KO + NAM (n = 20), OHT + NAM (n = 16), and KO + OHT (n = 20) groups. **(B)** RGC density across groups showed a significant decline in RGCs in the KO + OHT + NAM group (n = 6, p = 0.0173). There were no other statistical differences despite the decline in the KO + NAM (n = 6) and the OHT + NAM (n = 6) groups compared to the naïve NAM group (n = 6). **(C)** IOP was significantly higher in the OHT + NAM and KO + OHT + NAM groups from day 14–28 (****p < 0.0001, by repeated measures ANOVA). **(D)** Nicotinamide treatment resulted in no statistical differences in the Final PERG amplitude for the KO and KO + NAM group; however, there was a significant decline in Final PERG amplitude KO + NAM group compared to NAM alone (*p = 0.0461). Naive and KO PERG amplitudes from Figure 3 were included here for ease of comparison, with Final PERG amplitude in the KO mice significantly lower than Initial (^##^p = 0.0057). **(E)** Each of the Final PERG amplitudes was significantly lower than Initial for all of the OHT groups: OHT (^##^p = 0.0031); KO + OHT (^###^p = 0.0003); OHT + NAM (^##^p = 0.0015); and KO + OHT + NAM (^#^p = 0.0153). Initial PERG amplitude was significantly lower in OHT *versus* OHT + NAM (*p = 0.0324). Final PERG amplitude for OHT was significantly lower than OHT + NAM (*p = 0.0424). Final PERG amplitude for KO + OHT was significantly lower than OHT + NAM (**p = 0.0013) and KO + OHT + NAM (****p < 0.0001). Final OHT PERG amplitude is also significantly lower than KO + OHT + NAM final PERG amplitude (**p = 0.004). PERG and VEP all had the same n values: NAM n = 10, KO + NAM n = 20, OHT + NAM n = 16, and KO + OHT + NAM n = 20. There were no significant differences observed in PERG latency across groups (data not shown). **(F)** Amplitude of VEP was significantly reduced in the KO + OHT + NAM group (**p = 0.0013) when comparing Initial and Final measures. **(G)** NAM treatment resulted in a reduction in VEP latency for the KO + NAM group compared to KO alone (*p = 0.0473). There was a significant decrease in Final VEP latency in the OHT + NAM group (*p = 0.0142), and a significant difference in the Final VEP latency for the KO + NAM *versus* the OHT + NAM groups (*p = 0.0109).

Nicotinamide treatment has been demonstrated to overcome glaucoma-associated stressors as well as oxidative phosphorylation disruption through rotenone (Tribble et al., 2021). Since we have previously documented significant shifts in the complement of substrate transporters after glaucoma, we reasoned nicotinamide (NAM) may be capable of overcoming the energetic challenges presented by MCT2 KO, particularly if NAD + could facilitate TCA cycle function if carbon sources shift. In this second part of the study, mice were treated with 1 week of NAM prior to the induction of OHT, then for 4 weeks during OHT. Figure 7A shows immunohistochemistry for RBPMS in whole mount retina for each of the treatment groups. No significant change in RGC density was observed with the KO + NAM and OHT + NAM groups, but a statistically significant decline in RGC density was observed with the KO + OHT + NAM group (Figure 7B p = 0.0173). IOP was significantly higher in the OHT + NAM and KO + OHT + NAM groups from day 14 (Figure 7C). Nicotinamide did not mitigate the impact of MCT2 KO on PERG amplitude (compare black KO to red KO + NAM bars, Figure 7D) since there was no statistical difference between final KO PERG and final KO + NAM PERG amplitude. The final NAM PERG amplitude was also significantly higher than the KO + NAM PERG amplitude (p = 0.0461), indicating that the KO appears to decrease PERG amplitude despite NAM treatment. Note we include the Naive and KO data in this panel as presented in Figure 4 for ease of comparison. Final PERG amplitude was significantly lower than Initial for all of the OHT groups in Figure 7E (OHT, p = 0.0031; KO + OHT, p = 0.0003; OHT + NAM, p = 0.0015; and KO + OHT + NAM, p = 0.0153). In addition, initial PERG amplitude was statistically different from OHT *versus* OHT + NAM (p = 0.0324). Final PERG amplitude in OHT was statistically lower than final PERG amplitude in OHT + NAM (p = 0.0424); further significant comparions included final PERG amplitudes for KO + OHT *versus* OHT + NAM (p = 0.0013), OHT *versus* KO + OHT + NAM (p = 0.004), and KO + OHT *versus* KO + OHT + NAM (p < 0.0001). No significant differences were observed in PERG latency (data not shown). For VEP, NAM treatment was not sufficient to overcome the impact of the KO + OHT, resulting in a significant decline in VEP amplitude in the KO + OHT + NAM group (p = 0.0013, Figure 7F). VEP amplitude for Initial and Final measurements did not vary for any other groups. In contrast to part one of the study which resulted in no differences in VEP latency, NAM treatment resulted in a reduction in VEP latency for the KO + NAM group compared to KO alone (p = 0.0473, Figure 7G). There was a significant decrease in Final VEP latency in the OHT + NAM group (p = 0.0142), and a significant difference in the Final VEP latency for the KO + NAM *versus* the OHT + NAM groups (p = 0.0109, Figure 7G).

Similarly to part one of the study, we also evaluated gene expression for signs of a metabolic shift after treatment with NAM. No statistical differences in transcript expression for Slc2a3 (Glut3), Slc16a7 (MCT2), glutamate dehydrogenase, phosphofructokinase, lactate dehydrogenase-b, or mitochondrial pyruvate carrier were observed across the NAM groups, regardless of KO or OHT (Supplementary Figure S4A-F).

## 4 Discussion

Conditionally knocking out MCT2 in mouse RGCs to ascertain if MCT2 was necessary and sufficient for RGC survival and function revealed that loss of MCT2 did not impact RGC density but reduced RGC function as measured by PERG amplitude. MCT2 is therefore not necessary for short-term RGC survival, but its loss, which impacts RGC access to energy substrates, reduced RGC signaling. In response to MCT2 KO, RGCs upregulated other substrate transporters, including GLUT3, MCT1, and MCT4. By increasing the means by which RGCs could obtain glucose and pyruvate, lactate, and ketone bodies, RGC density was preserved. In anticipation of metabolic stress that might result from MCT2 KO, we investigated whether nicotinamide supplementation could assist with maintaining RGCs. Nicotinamide has previously been shown to be neuroprotective in the context of glaucoma in mice (Tribble et al., 2021; Boodram and Lim, 2024) and to preserve vision in glaucoma patients (Shukla et al., 2025). If MCT2 were necessary for RGC survival/function, we would expect that the loss of carbon entry into the TCA cycle as a result of the MCT2 KO would ensure nicotinamide would have no impact. Here, the nicotinamide intervention did not preserve RGC number in the KO + OHT group, though it slightly improved RGC function after OHT and in the MCT2 KO after OHT as measured by PERG amplitudes.

### 4.1 Our conditional knockout model eliminated MCT2 in the RGCs

MCT2, a transporter of lactate, pyruvate, and ketone bodies, is preferentially expressed on neurons such as the RGCs (Simpson et al., 2007). We have previously demonstrated that MCT2 is lost prior to glaucoma progression and that its overexpression was neuroprotective for RGCs (Harun-Or-Rashid et al., 2020). In this study, we used a conditional knockout of MCT2 so that the CNS would develop normally, with MCT2 integrated into metabolic pathways prior to its removal. The Thy1 promoter ensured RGC specificity because they express the Thy1 (CD90) glycoprotein (Huang et al., 2006). We confirmed the KO through immunolabeling for MCT2, *in situ* hybridization in the GCL, and qRT-PCR of isolated RGCs. The decrease in MCT2 immunolabel after OHT corroborates previously published data (Harun-Or-Rashid et al., 2018). The decline in MCT2 transcript in the MCT2 KO further confirms the knockout. The chaperone protein for MCT2, embigin (Felmlee et al., 2020), had a concomitant decline with MCT2 downregulation, an anticipated result given that embigin is required for efficient MCT2 trafficking to the plasma membrane (Ovens et al., 2010). Interestingly, MCT2 protein increased in the retina overall with KO and OHT, suggesting upregulation in other neurons. However, qRT-PCR results did not show MCT2 transcript upregulation in whole retina. Transporters have protein half-lives of hours to days (Dorrbaum et al., 2018), reducing any expectation to observe a good correlation between mRNA and protein. For example, anti-sense oligonucleotides used to eliminate MCT2 mRNA in hippocampal neurons resulted in MCT2 protein downregulation within hours (Suzuki et al., 2011).

### 4.2 MCT2 KO alone impacted RGC function but not RGC density

The astrocyte neuron lactate shuttle hypothesis posits that astrocytes release lactate as an end product of their glycolysis, which is taken up by RGCs via the MCT2 and used to meet their metabolic needs (Muraleedharan et al., 2020; Bonvento et al., 2005; Bhatti and Frostig, 2023). Even though neurons are capable of using glucose to meet their metabolic needs directly, recent studies have shown that in healthy animals, neurons under high levels of activity will opt for lactate (Dembitskaya et al., 2022). This knowledge, along with the previously observed decline in MCT2 in the RGCs in advance of glaucoma progression, and the neuroprotection conferred by MCT2 overexpression (Harun-Or-Rashid et al., 2020), led us to anticipate that MCT2 KO would be detrimental to RGC density and function. MCT2 KO alone had no impact on RGC density, suggesting that the RGCs met their metabolic needs sufficient for survival. The PERG amplitude data, however, showed a decline in RGC function after MCT2 KO. PERG amplitude represents inner retina and RGC electrical activity; amplitude reduction can be the result of RGC death or dysfunction (Porciatti et al., 2007). Since MCT2 KO did not result in measurable RGC loss, the lack of MCT2 likely caused RGC dysfunction, possibly from a reduction in the quantity of energy substrate available to the RGCs. MCT2 is required for the mRNA translation that underlies long-term memory formation in hippocampal neurons (Suzuki et al., 2011; Descalzi et al., 2019), indicating a specific role for glial lactate. Though long-lasting synaptic plasticity is primarily observed during circuit assembly in the retina (Wei et al., 2012), synaptic rewiring of the bipolar cell to RGC inputs can occur with disease (Tien et al., 2017; Fitzpatrick and Kerschensteiner, 2023). Loss of MCT2 may have negatively impacted the integrity of inner retinal circuits, thereby significantly reducing PERG amplitude in the MCT2 KO retina. As expected, OHT also significantly reduced PERG amplitude in WT retina, and in MCT2 KO retina. For VEP amplitude, only the KO + OHT group showed a significant decline from initial to final VEP measurement. MCT2 KO had no impact on the communication of RGCs to retinorecipient areas via the optic tract as assessed by VEP, but the addition of OHT to the KO significantly reduced signal arriving in the visual cortex. It is important to note that MCT2 loss was across the CNS since tamoxifen was systemically injected and Thy1 is expressed in many neurons. As a result, the stasis of VEP amplitude for MCT2 KO in WT mice implies a difference in the role of MCT2, or sufficient compensation by other transporters, in the retina compared to the optic projection.

### 4.3 MCT2 KO triggered upregulation of other metabolite transporters

Seeing no decline in RGC density after MCT2 KO suggested a potential compensatory mechanism for obtaining energy substrate by the RGCs. Both MCT1 and 4, monocarboxylate transporters observed primarily on endothelial cells and glia, respectively (Felmlee et al., 2020), were upregulated in RGCs with MCT2 KO. These transporters are not usually expressed by RGCs, and given their kinetics, could result in lactate transport out of the cell (Bergersen, 2007). However, we did observe overall MCT2 increase in the retina, particularly in the KO + OHT group, suggesting that neurons other than RGCs increased their expression of MCT2 and likely made use of the additional lactate potentially provided through MCT1 and MCT4. MCT2 is moderately expressed in the retina in amacrine and bipolar cells and minimally expressed in photoreceptors (Harun-Or-Rashid et al., 2020; Chandler et al., 2025). GLUT3, a glucose transporter expressed on neurons (Bélanger et al., 2011), including RGCs, was also upregulated after MCT2 KO. A pathway for increased glucose entry (through GLUT3) likely enabled preservation of RGC number because glucose can sustain ATP levels if fully metabolized through oxidative phosphorylation (Hall et al., 2012). Transcript levels of MCP1, the mitochondrial pyruvate carrier, did not differ in the retina and across groups, suggesting no shift in the balance of metabolism retina-wide. Ultimately, compensatory transporter upregulation was sufficient for RGC preservation, but not enough to protect RGC function as determined by the significant loss of PERG amplitude in the MCT2 KO retina.

We also measured ATP in whole retina, finding no statistical differences across groups. Similarly, immunolabeling for the P2X7 receptor, an ATP-gated cation channel that has been shown to be active in glaucoma and can drive inflammatory signaling (Lu et al., 2017), did not vary across groups. We did observe a significant increase in TNFα immunolabeling in the OHT group, indicating increased immune reactivity and presence of a cytokine that has been implicated in glaucoma pathogenesis (Kuchtey et al., 2010). ATP is extremely short-lived as a signaling molecule, and P2X7 receptor is only active at high ATP concentration (Beckel et al., 2014), so we did not expect to catch changes in levels. The low TNFα immunolabeling in the KO + OHT group seems to suggest that loss of MCT2 in the retina reduces inflammatory response. Metabolic shifts are a critical element of inflammatory response, as microglia and other immune cells use aerobic glycolysis upon cytokine stimulation (Soto-Heredero et al., 2020), producing lactate among other metabolites. The production of TCA cycle intermediates through aerobic glycolysis in these immune cells are important promoters of transcription factors like HIF-1α and NF-κB (Palsson-McDermott and O'Neill, 2013). If TNFα is produced as a result of OHT in microglia and Müller glia (Cueva Vargas et al., 2015), it is unlikely that the loss of MCT2 would calm inflammatory response since activated microglia are generating lactate that would join the lactate released by any glia expressing increased levels of MCT1 and MCT4, possibly resulting in detrimental extracellular acidification. RGCs express ASIC1a (Saafir et al., 2025), an acid sensing channel that can lead to increased intracellular Ca^++^ and cell death, particularly during ischemia (Xiong et al., 2004). We did not examine ASIC1a or acidification in the retina of our KO mice, but it could yield valuable insight.

### 4.4 Nicotinamide improved RGC function after OHT in MCT2 KO

Nicotinamide is the precursor of NAD^+^ in the salvage pathway, which is a key pathway to replenish NAD^+^ (Conlon and Ford, 2022), particularly in RGCs. NAD+ is a key electron acceptor in important metabolic processes such as glycolysis, the TCA cycle, and fatty acid oxidation. NADH, the reduced form of NAD^+^, drives ATP synthesis through oxidative phosphorylation (Xie et al., 2020). NAD^+^ loss is a driver of neurodegeneration (Gerdts et al., 2015; Gerdts et al., 2016; Gilley et al., 2015). Previous data has shown that supplementation with nicotinamide was neuroprotective (Tribble et al., 2021; Zhang et al., 2024). We speculated that nicotinamide supplementation could be sufficient to overcome metabolic insufficiency that would be the anticipated result of MCT2 KO because of its general maintenance of cellular energy levels (Fricker et al., 2018). MCT2 loss would primarily impact the TCA cycle and subsequent oxidative phosphorylation because it would diminish the availability of lacate and pyruvate. NAD + signaling does not directly impact MCT2 activity, but its role as a cofactor for glycolysis, the TCA cycle, and oxidative phosphorylation allows it to facilitate utilization of transported metabolic substrates. Since NAD+ is a major electron acceptor in glycolysis, glucose transport allows the cell to make more ATP from glucose and potentially overcome the impact of knocking out MCT2. The observed upregulation in GLUT3 in RGCs would enable glucose uptake, with options for ATP production through glycolysis or through to oxidative phosphorylation. NAD + would facilitate either of these scenarios. MCT2 KO alone resulted in a decline in RGC function, which was not mitigated by NAM supplementation. NAM was able to improve RGC survival after OHT, but it did not offer neuroprotection to RGCs subjected to OHT in which MCT2 was also eliminated. NAM also did not preserve PERG amplitude in the OHT or KO + OHT groups. Timing, dosing, and disease context are likely important determinants for the success of nicotinamide supplementation. For example, in a rat model of Parkinson’s disease, nicotinamide dose-dependently exacerbated dopaminergic neuron degeneration (Harrison et al., 2019).

### 4.5 Conclusion

This study examined whether MCT2 was necessary and sufficient for RGC survival and function. We determined that absence of MCT2 could be managed by RGCs through compensatory upregulation of other monocarboxylate and glucose transporters, which maintained RGC density but nevertheless diminished RGC PERG amplitude. Loss of MCT2 negatively impacted RGC electrical signaling. NAM supplementation was able to improve PERG amplitudes after OHT, with or without MCT2 KO. Overall, the data suggests that maintenance of MCT2 is important for RGC function.

## Data Availability

The original contributions presented in the study are included in the article/Supplementary Material, further inquiries can be directed to the corresponding author.
