# Supplementary material for: Compensatory transporter upregulation facilitates retinal ganglion cell survival in glaucoma after MCT2 elimination
Source: Front Cell Dev Biol. 2026 Apr 29;14:1805959. doi: 10.3389/fcell.2026.1805959 (PMC13168206; doi:10.3389/fcell.2026.1805959)
Supplement: Supplementary file 1 [file DataSheet1.docx]

**Compensatory transporter upregulation facilitates retinal ganglion cell survival in glaucoma after MCT2 elimination**

Supplementary Data


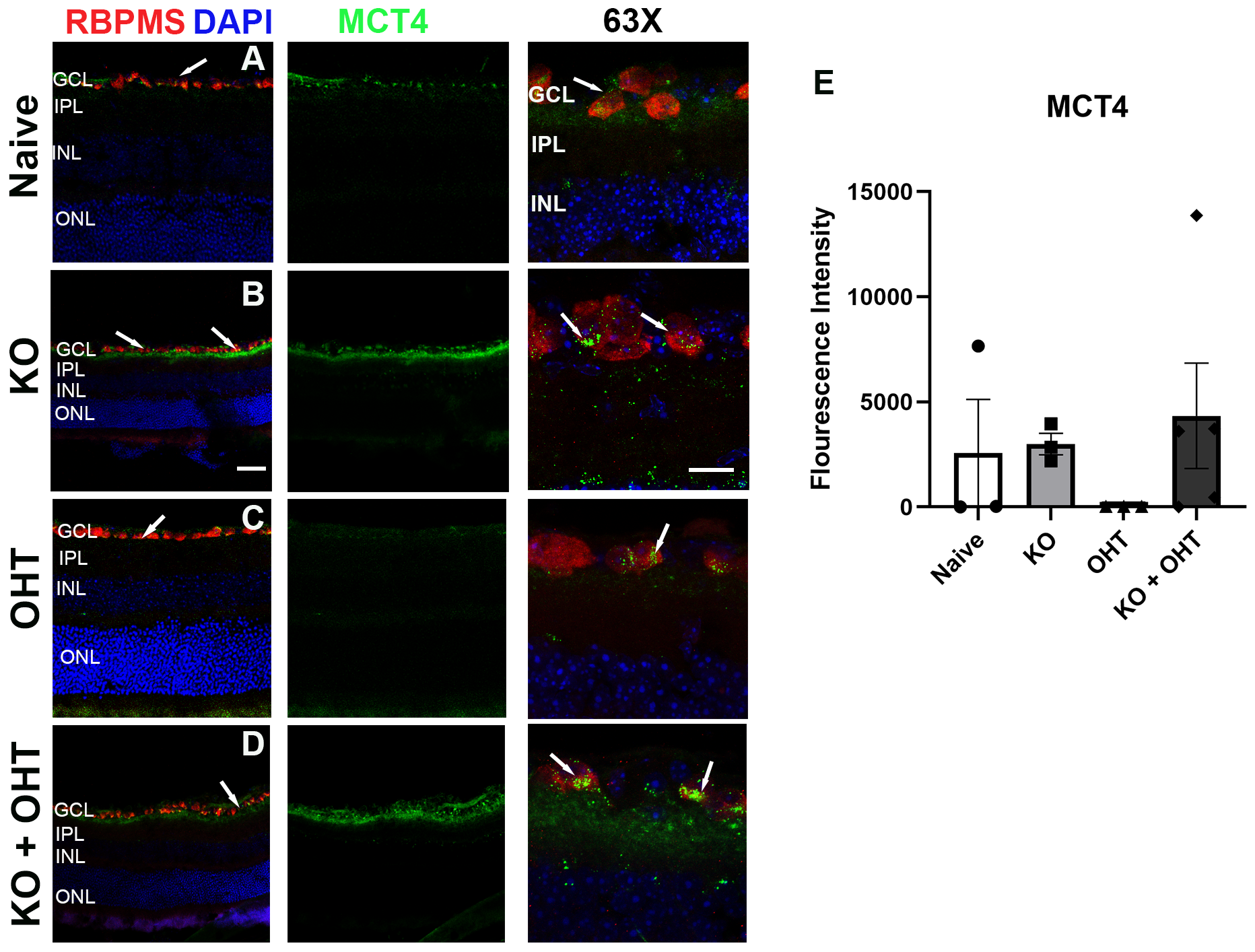


**Supplementary Figure 1**. Immunolabeling for MCT4. **A**. MCT4 in the Naive group was associated with RBPMS-positive RGCs (arrow on right). In the sections from KO retina (**B**), the MCT4 immunolabel was more extensive, including into the adjacent areas of the IPL (arrow in B). These inner portions of IPL are the dendritic arbors of ON-RGCs. **C**. After OHT, MCT4 immunolabel was absent in the IPL and extremely low in the GCL. **D**. Knockout of MCT2 with OHT promoted MCT4 immunolabel increase in the GCL and inner IPL, the dendritic arbors for ON-RGCs. Scale bars=50 µm **E.** The immunolabeling quantification showed no statistically significant differences among the experimental groups: Naïve (n=3), KO (n=3), OHT (n=3), and KO+OHT (n=4)- despite high variation across groups.


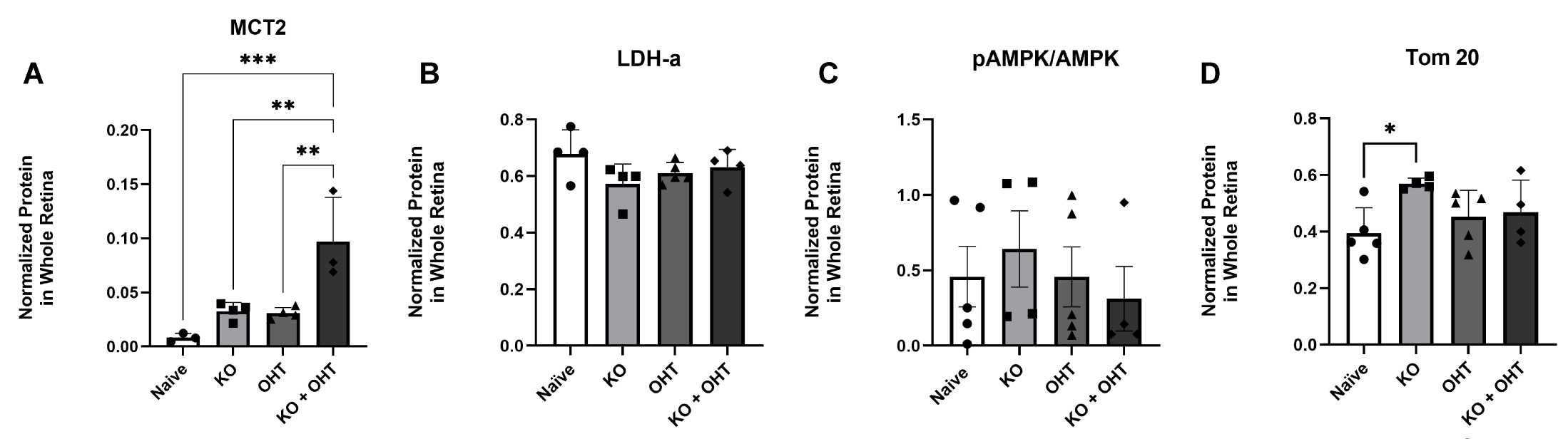


**Supplementary Figure 2**. Protein analysis using whole retina lysates. **A**. The KO + OHT (n=3) group had significantly more MCT2 protein than the Naïve (n=3, p=0.0010), KO (n=4, p= 0.0063), and OHT (n=4, p=0.0051) groups. **B-C**. No statistically significant differences in protein amounts were observed across experimental groups for LDH-A (B), Naïve n=4,KO n=4, OHT n=5 and KO+OHT n=4 and the ratio of pAMPK to AMPK (C) Naïve n=5, KO n=4, OHT n=5 and KO+OHT n=4. **D**. The mitochondrial marker Tom 20 was significantly higher in the KO (n=4) group than the Naïve (n=5) group (*p=0.0441) but not in the OHT (n=5) and KO+OHT (n=4).


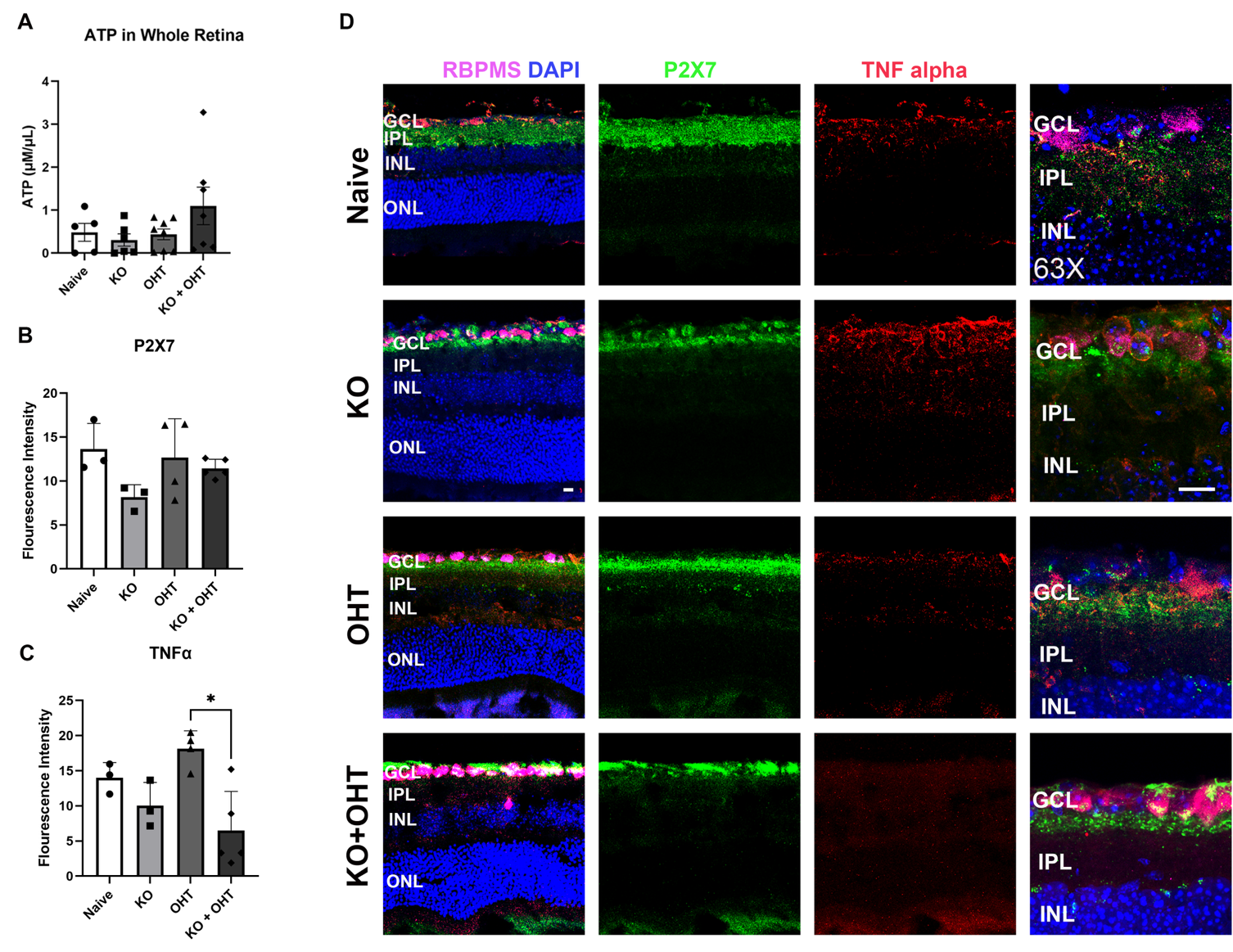


**Supplementary Figure 3**. ATP and P2X7 receptor Analysis. **A**. ATP measured in retinae from Naïve (n=5), KO (n=6), OHT (n=8), and KO+OHT (n=7) groups showed no significant differences across groups. **B**. Quantification of mean fluorescence intensity of P2X7 receptor immunolabeling as shown in green (D). No significant differences were seen for P2X7 in the GCL among Naïve (n=3), KO (n=3), OHT (n=4) and KO+OHT (n=5). **C**. Quantification of TNFα immunolabeling (mean fluorescence intensity) as shown in red (D). The OHT (n=3) group had significantly more TNFα immunolabel than the KO + OHT (n=3) group in the GCL layer (*p=0.0247) but not the Naïve (n=3) and KO (n=3). **D**. Immunolabeling for RBPMS (magenta), P2X7 (green), and TNFα (red) across the experimental groups. P2X7 was observed throughout the inner retina, including IPL and GCL. TNFα immunolabeling was also observed in inner retina, primarily in inner IPL and GCL. There were subtle differences in immunolabel across groups. Scale bars=50µm


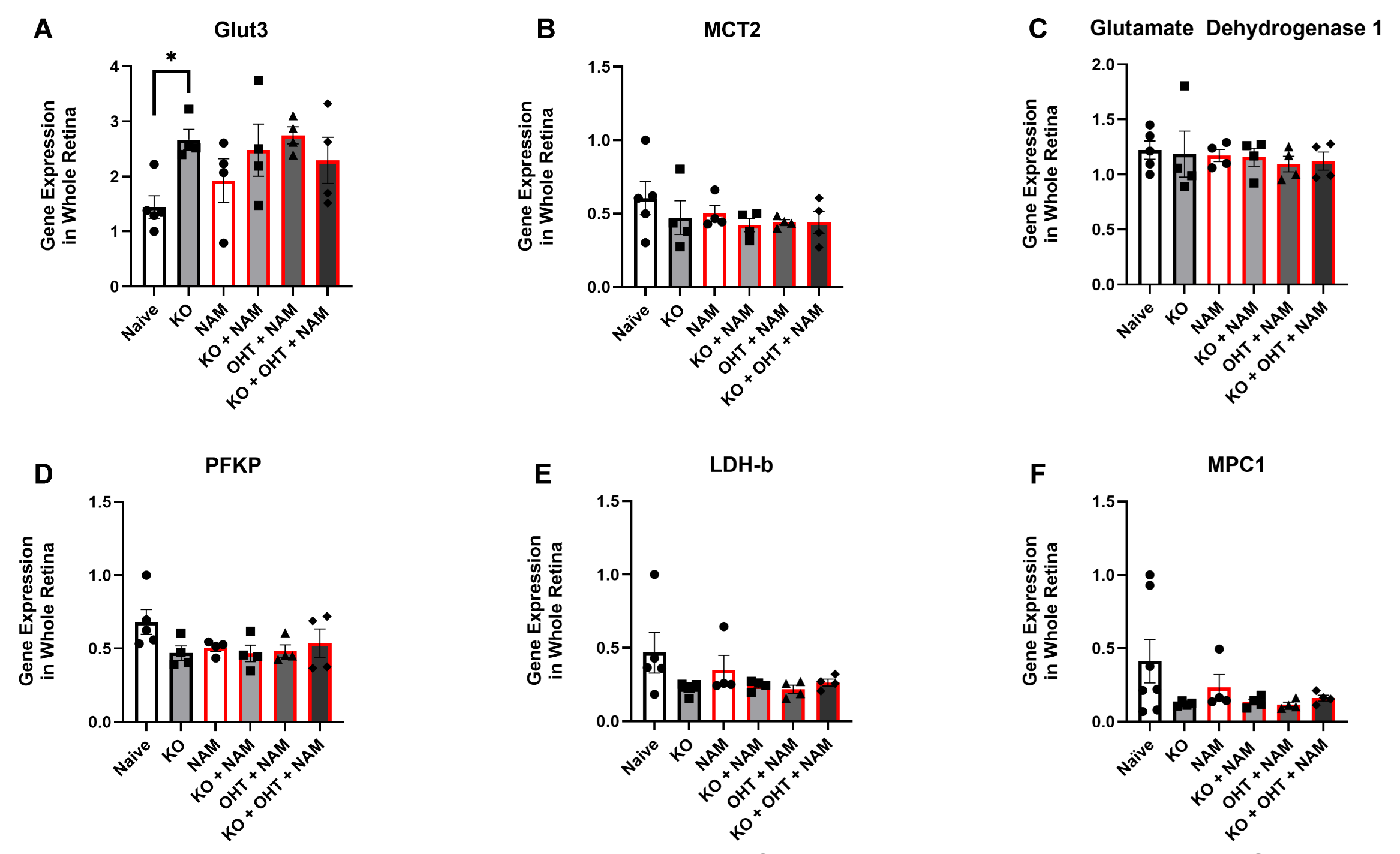


**Supplementary Figure 4.** Quantitative RT-PCR analysis of transcripts from whole retinae for the NAM treatment study. **A**. Glut3 transcript was significantly increased in the KO group, which had significantly more as compared to the Naïve group (*p<0.05). **B-F**. There were no statistical differences in gene expression across the experimental groups for monocarboxylate transporter 2 (MCT2) (B), Glutamate Dehydrogenase 1 (C), Phosphofructokinase (PFKP) (D), lactate dehydrogenase B (LDH-b) (E), and mitochondrial pyruvate carrier (MPC1) (F). Glut3, MCT2, Glutamate Dehydrogenase 1, PFKP, and LDH-b and MPC1 had n=4 across all the NAM treatment groups.
